# The SGLT2 inhibitor empagliflozin reduces tissue sodium content in patients with chronic heart failure: results from a placebo-controlled randomised trial

**DOI:** 10.1007/s00392-022-02119-7

**Published:** 2022-10-26

**Authors:** Julie Kolwelter, Dennis Kannenkeril, Peter Linz, Susanne Jung, Armin M. Nagel, Agnes Bosch, Christian Ott, Peter Bramlage, Lisa Nöh, Mario Schiffer, Michael Uder, Stephan Achenbach, Roland E. Schmieder

**Affiliations:** 1 Department of Nephrology and Hypertension, University Hospital Erlangen, Friedrich-Alexander University Erlangen-Nuremberg (FAU), Ulmenweg 18, 91054 Erlangen, Germany; 2 Department of Cardiology, University Hospital Erlangen, Friedrich-Alexander University Erlangen-Nuremberg (FAU), Erlangen, Germany; 3 Institute of Radiology, University Hospital Erlangen, Friedrich-Alexander University Erlangen-Nuremberg (FAU), Erlangen, Germany; 4 Division of Medical Physics in Radiology, German Cancer Research Center (DKFZ), Heidelberg, Germany; 5 Department of Nephrology and Hypertension, Paracelsus Medical University, Nuremberg, Germany; 6 Institute for Pharmacology and Preventive Medicine, Cloppenburg, Germany

**Keywords:** Empagliflozin, SGLT2 inhibitor, Sodium, Chronic heart failure, Tissue sodium content, Magnetic resonance imaging

## Abstract

**Introduction:**

Sodium-glucose co-transporter 2 (SGLT2) inhibitors have cardiovascular protective properties in addition to the metabolic effects and represent a cornerstone of treating patients with chronic heart failure (CHF). We hypothesised that empagliflozin reduces tissue sodium content in patients with CHF.

**Methods:**

In a double-blind, randomised (2:1), placebo-controlled, parallel-group, clinical trial, 74 patients with NYHA class II–III CHF and an ejection fraction of 49% or less received empagliflozin 10 mg once daily or placebo for 3 months. In each patient, tissue sodium content of the lower leg was assessed non-invasively by sodium-MRI (^23^Na-MRI) at baseline, after 1 and 3 months of treatment.

**Results:**

After 1 and 3 months treatment with empagliflozin (*n* = 48), a significant decrease in skin sodium content was observed (1 month: 22.8 ± 6.1 vs. 21.6 ± 6.0 AU, *p* = 0.039; 3 months: 22.9 ± 6.1 vs. 21.6 ± 6.1 AU, *p* = 0.013), while there was no change in muscle sodium and muscle water content. In direct comparison, the change in skin sodium content between baseline and 3 months was − 1.3 ± 3.5 AU in the empagliflozin group versus 0.6 ± 3.5 AU in the placebo group (*p* for between-group difference = 0.022). No significant difference regarding change in muscle sodium and in muscle water content was observed after 3 months treatment between the two groups.

**Conclusion:**

This trial showed a significant decrease in skin sodium content after 1 and 3 months of treatment with empagliflozin. The decrease in skin sodium content may reflect a decrease in subclinical micro-oedema or/and in non-osmotic bound tissue sodium, both reported to impair left ventricular function.

**Trial registration number:**

NCT03128528 (http://www.clinicaltrials.gov).

**Trial registration date:**

25th April 2017.

**Supplementary Information:**

The online version contains supplementary material available at 10.1007/s00392-022-02119-7.

## Introduction

Sodium glucose co-transporter 2 (SGLT2) inhibitors were originally developed to treat hyperglycaemia in patients with type 2 diabetes (T2D) and became further a cornerstone of treating patients with chronic heart failure (CHF) [1–4]. The precise mechanisms of the cardiovascular protective properties of SGLT2 inhibitors are only partially understood [5–7].

For years, excess of intravascular sodium (e.g. due to excessive sodium intake) has been assumed to be always accompanied by water retention, leading to increased plasma volume and higher cardiac output. However, knowledge of sodium metabolism has progressed and the 2-compartment model has been replaced by the 3-compartment model, in which excess sodium is bound non-osmotically to negative charged glycosaminoglycans present in the extracellular space, mainly in the skin, endothelial surface layer, muscle and bone [8–11]. After intravenous hypertonic saline infusion in healthy individuals on a low-sodium diet, only 47% of the expected sodium excretion was retrieved in the collected urine, suggesting an osmotically inactive accumulation of a significant amount of sodium in the tissue [12].

Recent developments in sodium-MRI (^23^Na-MRI) technique have facilitated the non-invasive investigation of tissue sodium content in human tissues [13, 14]. Increased tissue sodium content has been found in several populations with cardiovascular disease including patients with refractory hypertension, T2D, chronic kidney disease, left ventricular hypertrophy and acute heart failure [15]. Interestingly, in patients with primary aldosteronism, increased sodium content has also been measured in the myocardium [16].

In the current trial, we focused on patients with chronic heart failure (CHF) and mid-range (HFmrEF) or reduced (HFrEF) ejection fraction in stable euvolemic condition. Our aim was to analyse the changes of tissue sodium content in these patients after 1 and 3 months of treatment with empagliflozin from baseline and compared to placebo.

## Material and methods

### Trial design

The trial was a prospective, investigator initiated, double-blind, randomised, placebo controlled, parallel-group clinical trial, performed at the Clinical Research Centre of the University Hospital Erlangen, Germany. We recruited patients with HFrEF or HFmrEF, according the 2016 ESC guidelines definition, from the University outpatient clinic as well as through newspaper advertisements. All patients had to be in a stable condition with New York Heart Association class (NYHA) II–III.

**Fig. 1:**
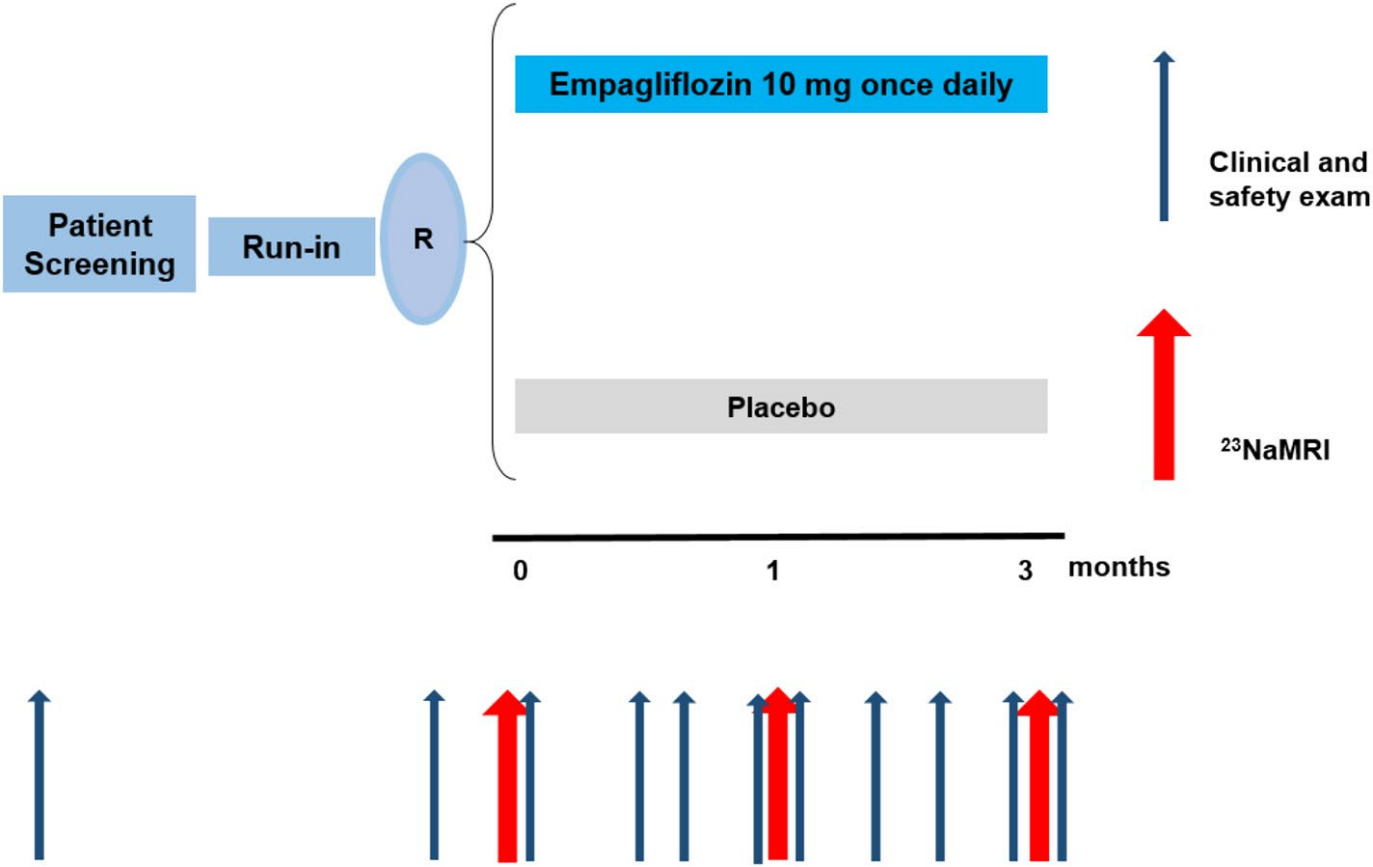
Trial design: summary of study visits and tests performed in each visit

Each patient entered, after being screened for eligibility, a 2-weeks run-in phase where, if necessary, change of medication to meet the patient’s clinical condition or additional diagnostics (e.g. echocardiogram) took place. After the 2-weeks run-in phase, being naive to or off any SGLT2 inhibitor for at least 10 weeks, patients underwent the first ^23^Na-MRI exam of the lower leg and clinical parameters were assessed. Patients were then consecutively randomised (2:1) to either empagliflozin 10 mg once daily or placebo (with no difference in shape, colour or taste of the pill) for 3 months. After 1 and 3 months, ^23^Na-MRI parameters, clinical parameters, vascular parameters as well as flow mediated vasodilatation (FMD) measurements were assessed (Fig. 1). The results on vascular parameters under 24-h daily conditions as well as under laboratory conditions were already previously published [17].

After ^23^Na-MRI acquisition, every data set was evaluated offline by two independent trained and certified investigators under the supervision of the most experienced senior investigator of the Institute of Radiology. All investigators were blinded with respect to any clinical data, including the allocation to the treatment group and time of the obtained ^23^Na-MRI measurements.

### Trial population

Patients between 18 and 85 years of age and who had a CHF with an ejection fraction below 40% (HFrEF) or an ejection fraction between 40 and 49% as well as a N-terminal pro-brain natriuretic peptide (NTproBNP) level > 125 pg/ml and at least one structural abnormality of the left atrium or ventricle (HFmrEF), in stable conditions, were included. Individuals were excluded, if they had any other form of diabetes than T2D, were treated with insulin or more than one oral anti-diabetic drug or any SGLT2 inhibitor within the last 10 weeks prior to the screening visit or were treated with loop diuretics (above furosemide > 80 mg/day, torasemide > 40 mg/day or piretanide > 6 mg/day), had an uncontrolled diabetes (glycated haemoglobin (HbA1c) ≥ 10% or fasting-plasma glucose level > 240 mg/dl) or uncontrolled blood pressure (BP ≥ 180/100 mmHg), had an estimated glomerular filtration rate (eGFR) < 30 ml/min/1.73 m^2^ or had CHF NYHA class IV within the last 3 months.

### Endpoints

The primary endpoint of the trial was to analyse the effect of 3 months treatment with empagliflozin on change in tissue (skin, muscle, bone marrow) sodium content compared to placebo. Secondary endpoints were the effect of 1 month treatment with empagliflozin on change in tissue sodium content compared to placebo as well as the effect of 1 and 3 months treatment with empagliflozin compared to baseline.

### Clinical parameters

At the first visit, all demographic data were recorded. At the randomisation visit, a fasting blood sample was taken in order to determine the NTproBNP level, serum sodium level, HbA1c and fasting plasma glucose as well as other biochemical safety parameters (e.g. serum creatinine, eGFR). Twenty-four-hour urine was collected to assess the urine volume, sodium as well as glucose excretion over 24 h. The 24-h urine collections were primarily frozen in order to keep the double-blind aspect of the trial maintained, the analyses were performed after the end of the trial.

Office BP and heart rate was assessed after 5 min of rest according to guidelines [18]. All biochemical parameters and ^23^Na-MRI parameters were assessed at baseline, after 1 and 3 months. Any adverse events occurring during the trial were recorded at each visit.

### Body composition measurements

Fluid status of the patients was assessed by a whole-body bioimpedance spectroscopy (Body Composition Monitor (BCM), Fresenius Medical Care, Bad Homburg, Germany) at baseline, after 1 and 3 months. From the impedance data and additional clinical parameters, extracellular water, intracellular water and total water was calculated by the equations proposed by Moissl [19].

Fluid status is represented by overhydration (OH in litre), based on a three-compartment model developed by Chamney [20]. OH corresponds to the difference between the BCM detected extracellular water in the tissue and the predicted water content based on physiologic models under euvolemic conditions.

### ^23^Na-MRI measurements

In our trial tissue sodium, muscle water and muscle fat content in the lower leg were specially measured with a 3.0 T clinical MR system (Magnetom Skyra, Siemens Healthineers, Erlangen, Germany) using a custom-made transit/receive sodium RF birdcage knee coil (32.6 MHz, Stark Contrast, Erlangen, Germany). Reliability as well as accuracy have been shown previously [8].

**Fig. 2:**
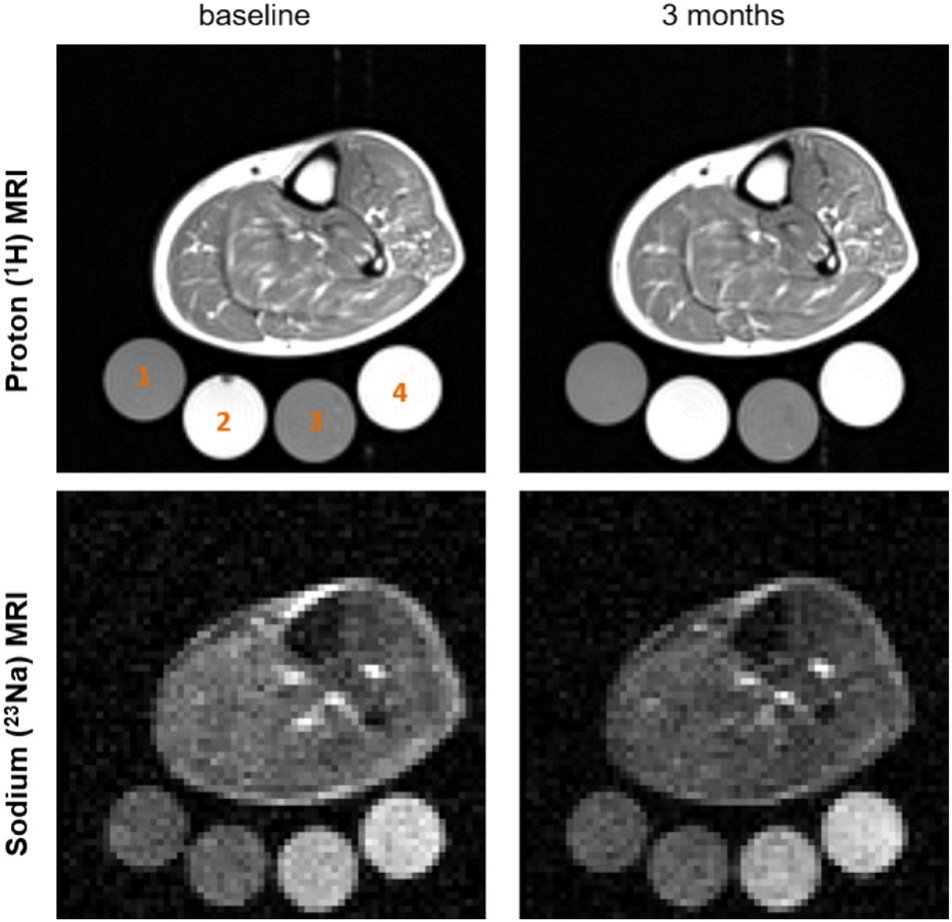
Sample of ^1^H-MRI and ^23^Na-MRI image acquisition of the lower leg at baseline (left) and after 3 months treatment with empagliflozin (right). The calibration tubes 1–4 are placed in the phantom holder underneath the calf. Calibration tubes – 1: 20 mmol Nacl + 5% agarose gel, 2: 20 mmol NaCl, 3: 40 mmol NaCl + 5% agarose gel, 4: 40 mmol NaCl

In brief, patients were in supine position with the thickest part of their left or right calf muscle at the centre of the sodium RF birdcage knee coil with calibration tubes placed in a phantom holder underneath the calf and included in the field of view. Partial volume effects and short relaxation times of sodium ions result in a more than threefold underestimation of measured sodium concentrations and in order to reflect this, the signal intensities are reported in arbitrary units (AU). Proton and sodium images were then acquired (Fig. 2). Descriptions of the different used sequences are reported in the Online Resource.

Interrater variability calculations showed according to Bland Altman [21] a close agreement between the two investigators with a mean of the differences (± standard deviation (SD)) of − 0.13 ± 0.78 AU for skin sodium content and of 0.10 ± 0.29 AU for muscle sodium content. The coefficient of determination (*R*^2^) was 0.98 and 0.99, respectively.

### Statistical analysis

Statistical analysis was performed using SPSS Statistics 28. The sample size was calculated for the primary endpoint using data acquired during a previous trial, the SD of change in skin sodium content was 4.17 AU in patients with T2D [22]. In order to detect a change of 2.9 AU after 3 months of empagliflozin as opposed to placebo, for *α* = 0.05 and *ß* = 0.80, the required number of patients was calculated to be 75 leading to an overall sample size of *N* = 84 including patients respective to a 12% drop-out rate.

All analyses of this trial were performed in patients presenting at least a ^23^NaMRI exam after randomisation visit and showing no major protocol violations (modified intention-to-treat approach). Normal distribution of the parameters was assessed by the Shapiro Wilk test. Continuous variables are presented as mean with SD if normally distributed and median (interquartile range) if non-normally distributed. Categorical variables are presented as frequency and percentage. The appropriate parametric and non-parametric tests were used to compare the continuous variables. *X*^2^-Test/Fisher’s exact test were used to compare categorical variables. Data from ^23^Na-MRI and patients clinical and laboratory characteristics were analysed by Student’s *t*-test for paired (baseline to 1 or 3 months) and unpaired (empagliflozin vs. placebo) samples, if normally distributed. Non-normally distributed parameters were analysed by Wilcoxon test and Mann–Whitney *U* test, respectively. Additionally, we examined the difference between the two groups (empagliflozin vs. placebo) over time by using the ANOVA analysis. Further, we added parameters most susceptible to influence skin sodium content such as treatment with mineralocorticoid receptor antagonist, sex, age, weight, systolic BP and ejection fraction as covariates in the ANCOVA model for the analysis of our primary objective. Correlations were assessed using Spearmann and Pearson correlation coefficient for non-normally and normally distributed variables, respectively. A two-sided *p* value < 0.05 was considered statistically significant.

## Results

### Trial population

A total of 87 patients were screened and 75 of them were randomised. The intention-to-treat population included 74 patients, 1 patient being excluded due to major protocol violation. The modified intention-to-treat population included 72 patients, 48 in the empagliflozin group and 24 in the placebo group. Two patients were excluded due to withdrawal of consent before the first ^23^Na-MRI analysis was performed (Online Resource Fig. 1). All patients were receiving appropriate treatment for heart failure with no change of the therapy regimen after the randomisation visit until the end of the trial period. Patients were in stable clinical condition with NYHA class II-III and showed no clinical signs of congestion such as macro-oedema, orthopnoea, crepitation or wheeze. Patient characteristics including medication are summarised in Table 1.

**Table 1:** Patient characteristics

Parameters	All (*n* = 74)	Empagliflozin (*n* = 48)	Placebo (*n* = 26)	*p* value
Age (years)	66.4 ± 8.9	67.7 ± 8.8	64.0 ± 8.8	0.076
Male sex (*n*, %)	62 (84)	39 (81)	23 (88)	0.522
Weight (kg)	89.0 ± 13.5	88.0 ± 13.5	90.9 ± 13.4	0.382
BMI (kg/m^2^)	29.0 ± 3.8	28.8 ± 3.9	29.5 ± 3.5	0.401
Office heart rate (bpm)	65.1 ± 11.4	65.3 ± 11.7	64.9 ± 11.0	0.883
Office systolic BP (mmHg)	122.2 ± 18.7	122.8 ± 19.7	121.0 ± 17.1	0.707
Office diastolic BP (mmHg)	72.3 ± 8.8	72.3 ± 9.0	72.2 ± 8.5	0.931
eGFR (ml/min/1.73 m^2^)	73.5 ± 17.6	74.1 ± 16.3	72.6 ± 19.9	0.731
Serum creatinine (mg/d)	1.04 ± 0.25	1.02 ± 0.23	1.09 ± 0.29	0.225
HbA1c (%)	5.9 ± 0.6	5.9 ± 0.6	5.9 ± 0.7	0.954
Fasting-plasma glucose (mg/dl)	101.5 ± 17.1	101.2 ± 15.1	102.0 ± 20.5	0.856
NTproBNP (pg/ml)	444 (247, 1064)	472 (293, 1165)	417 (209, 1064)	0.500
Left ventricular ejection fraction (%)	40 (35, 45)	43 (37, 45)	38 (28, 43)	0.060
HFmrEF (*n*, %)	44 (59.5)	32 (67)	12 (46)	
HFrEF (*n*, %)	30 (40.5)	16 (33)	14 (54)	
Cause of heart failure				0.679
Ischemic (*n*, %)	52 (70)	33 (69)	19 (73)	
Non-ischemic (*n*, %)	22 (30)	15 (31)	7 (27)	
Cardiovascular history				
Arterial hypertension (*n*, %)	57 (77)	35 (73)	22 (85)	0.253
Diabetes mellitus type 2 (*n*, %)	17 (23)	12 (25.0)	5 (19)	0.573
Atrial fibrillation (*n*, %)	32 (43)	21 (44)	11 (42)	0.905
Heart failure treatment				
AT1-receptor antagonist (*n*, %)	20 (27)	15 (31)	5 (19)	0.266
ACE-inhibitor (*n*, %)	35 (47)	19 (40)	16 (62)	0.071
Sacubitril/Valsartan (*n*, %)	12 (16)	5 (10)	7 (30)	0.743
Beta-blocker (*n*, %)	56 (76)	37 (77)	19 (73)	0.701
Calcium channel blocker (*n*, %)	13 (18)	8 (17)	5 (19)	0.760
Mineralocorticoid receptor antagonist (*n*, %)	41 (55)	24 (50)	17 (65)	0.204
Thiazide (*n*, %)	15 (20)	8 (17)	7 (27)	0.295
Loop diuretic (*n*, %)	29 (40)	19 (40)	10 (38)	0.972

Data are mean ± standard deviation or median (interquartile range)

*BMI* body mass index, *bpm* beats per minute, *BP* blood pressure, *eGFR* estimated glomerular filtration rate, *HbA1c* glycated haemoglobin, *NTproBNP* N-terminal prohormone of brain natriuretic peptide, *HFmrEF* heart failure with mid-range ejection fraction, *HFrEF* heart failure with reduced ejection fraction, *AT1-receptor* angiotensin 1 receptor, *ACE* angiotensin-converting enzyme

### Adherence

Adherence to trial medication (pill counting) as well as overall adherence was given with 99 ± 3%. All patients in the empagliflozin group presented significant urinary glucose excretion at 1 and 3 months follow-up visits. None of the patients in the placebo group showed urinary glucose excretion (no drop-ins).

### ^23^Na-MRI parameters

Compared to baseline, patients in the empagliflozin group showed a significant reduction of skin and bone marrow sodium content after 1 month of treatment (22.8 ± 6.1 vs. 21.6 ± 6.0 AU, *p* = 0.039; 3.0 ± 1.6 vs. 2.5 ± 1.4 AU, *p* = 0.010) and after 3 months of treatment (22.9 ± 6.1 vs. 21.6 ± 6.1 AU, *p* = 0.013; 3.1 ± 1.7 vs. 2.5 ± 1.4 AU, *p* = 0.013) (Online Resource Table 1, Table 2). Muscle sodium, muscle water and muscle fat content were not significantly different after 1 month and 3 months of treatment with empagliflozin compared to baseline. In the placebo group, neither of the tissue sodium parameters, muscle water or muscle fat content showed a significant change after 1 and 3 months compared to baseline.

**Table 2:** Changes in ^23^NaMRI parameters after 3 months of treatment with empagliflozin or placebo

Parameters after 3 months	Empagliflozin, change from baseline	*p* value empagliflozin vs. baseline	Placebo, change from baseline	*p* value placebo vs. baseline	Between-group difference	*p* value empagliflozin vs. placebo
Skin sodium content (AU)	− 1.3 ± 3.5	0.013	0.6 ± 3.5	0.384	− 2.1 ± 0.9	0.022
Muscle sodium content (AU)	− 0.3 ± 2.9	0.448	− 0.9 ± 3.0	0.169	0.5 ± 0.8	0.536
Muscle sodium content, inversion recovery sequence (AU)	− 0.1 ± 2.1	0.751	− 0.8 ± 2.6	0.141	0.7 ± 0.6	0.250
Muscle water content (ms)	0.5 ± 5.4	0.516	− 0.3 ± 4.5	0.732	0.8 ± 1.3	0.517
Muscle fat content (AU)	0.2 ± 2.1	0.481	0.4 ± 2.4	0.490	0.3 ± 0.6	0.790
Tibial bone marrow sodium content (AU)	− 0.6 ± 1.4	0.013	0.1 ± 1.1	0.825	− 0.6 ± 0.4	0.074

Data are mean ± standard deviation

*AU* arbitrary units

**Fig. 3:**
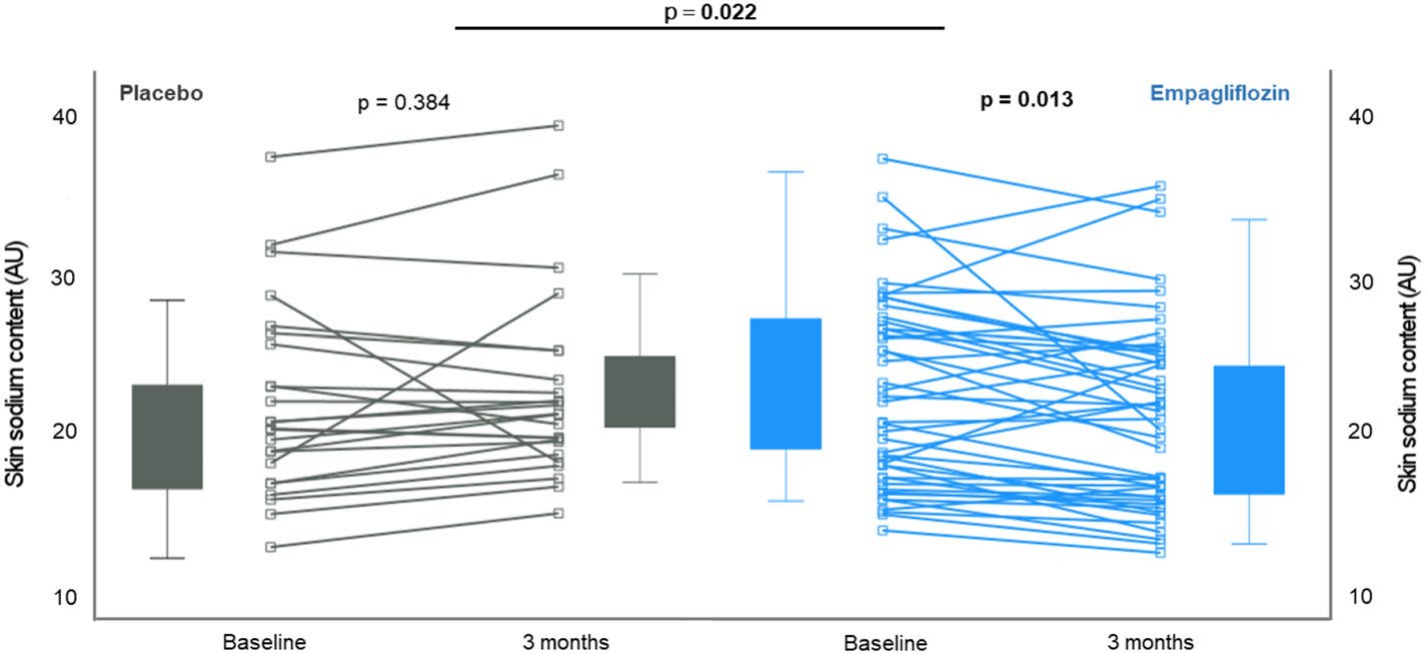
Primary endpoint: Change in skin sodium content after 3 months treatment with empagliflozin in the two groups—individual trajectories and boxplot showing the change from baseline to 3 months in each group

Comparing the two groups, the change in skin sodium content between baseline and 3 months was − 1.3 ± 3.5 AU in the empagliflozin versus 0.6 ± 3.5 AU in the placebo group (*p* for between-group difference = 0.022) (Fig. 3), whereas the change between baseline and 1 month was not significantly different between the 2 groups. After adjustment for potential confounders such as treatment with mineralocorticoid receptor antagonist, sex, age, weight, systolic BP and ejection fraction (ANCOVA), the difference regarding change in skin sodium content was still significant after 3 months treatment between the two groups.

No significant differences regarding the change in muscle sodium, muscle water or muscle fat content were observed after 1 and 3 months treatment with empagliflozin compared to placebo. In the inversion recovery sequence designed to estimate partly the amount of bound sodium ions, no differences were observed after 1 and 3 months of either treatment compared to baseline and between the two groups. The change in bone marrow sodium content between baseline and 3 months was − 0.6 ± 1.4 AU versus 0.1 ± 1.1 AU in the placebo group (*p* for between-group difference = 0.074).

**Fig. 4:**
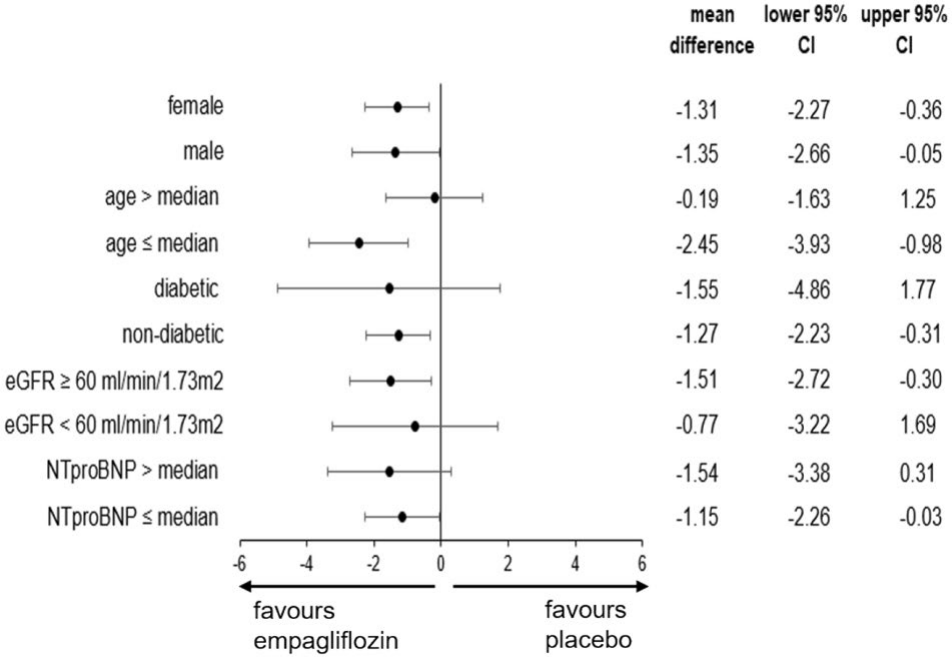
Forest Plot of subgroup analysis for the primary endpoint: change in skin sodium content after 3 months treatment with empagliflozin versus placebo. *eGFR* estimated glomerular filtration rate; mean difference = skin sodium content at 3 months minus skin sodium content at baseline, *NTproBNP* N-terminal prohormone of brain natriuretic peptide, *95% CI* 95% confidence interval, median age = 67 years; median NTproBNP = 444 pg/ml

Prespecified subgroup analyses according to gender (male/female), age (according to the median of age), diabetes status, chronic kidney disease stage (according to the eGFR < 60 ml/min/1.73 m^2^) or severity of CHF (according to the median of NTproBNP level) showed consistent results without any heterogeneity, as found in the whole trial population, namely a reduction of skin sodium content in the empagliflozin group after 3 months (Fig. 4).

### Clinical characteristics

In the empagliflozin group, there was a decrease in weight, BMI, eGFR and parameters of diabetic control, such as fasting plasma glucose and HbA1c, and an increase in haematocrit, serum creatinine and serum uric acid after 1 (Online Resource Table 2 and Table 3) and 3 months (Table 3, Online Resource Table 4). There were no differences for the same parameters in the placebo group. NTproBNP level as well as serum sodium level did not change under either condition compared to baseline as well as between the two groups after 1 and 3 months.

**Table 3:** Changes in clinical parameters after 3 months of treatment with empagliflozin or placebo

Parameters after 3 months	Empagliflozin change from baseline	*p* value empagliflozin vs. baseline	Placebo change from baseline	*p* value placebo vs. baseline	Between-group difference	*p* value empagliflozin vs. placebo
Weight (kg)	− 0.8 ± 1.8	0.002	0.5 ± 2.3	0.329	− 1.3 ± 0.5	0.010
BMI (kg/m^2^)	− 0.3 ± 0.6	0.003	0.2 ± 0.7	0.310	− 0.4 ± 0.2	0.011
Office SBP (mmHg)	− 5.0 ± 16.8	0.043	1.9 ± 11.9	0.437	− 7.0 ± 3.8	0.074
Office DBP (mmHg)	− 1.6 ± 7.8	0.169	− 1.2 ± 7.6	0.448	− 0.4 ± 1.9	0.842
Office heart rate (bpm)	1.1 ± 13.0	0.560	− 0.2 ± 10.6	0.919	2.0 ± 3.8	0.600
OH (l)	− 0.5 ± 1.3	0.016	− 0.5 ± 2.7	0.377	0.04 ± 0.5	0.935
OH/ECW (%)	− 2.5 ± 6.4	0.013	− 2.3 ± 11.7	0.372	− 0.2 ± 2.2	0.936

Data are mean ± standard deviation

*BMI* body mass index, *SBP* systolic blood pressure, *DBP* diastolic blood pressure, *bpm* beats per minute, *OH* overhydration, *OH/ECW* ratio of overhydration and extracellular water

In the empagliflozin group, 24-h urine volume excretion as well as 24-h urine sodium excretion increased after 1 month treatment versus baseline and the 1-month increase of urine volume excretion was significantly greater than in the placebo group (*p* = 0.046). After 3 months of treatment with empagliflozin, the change in 24-h urine sodium excretion was no longer significant compared to baseline, whereas 24-h urine volume excretion remained increased (*p* = 0.019).

### Correlations

**Fig. 5:**
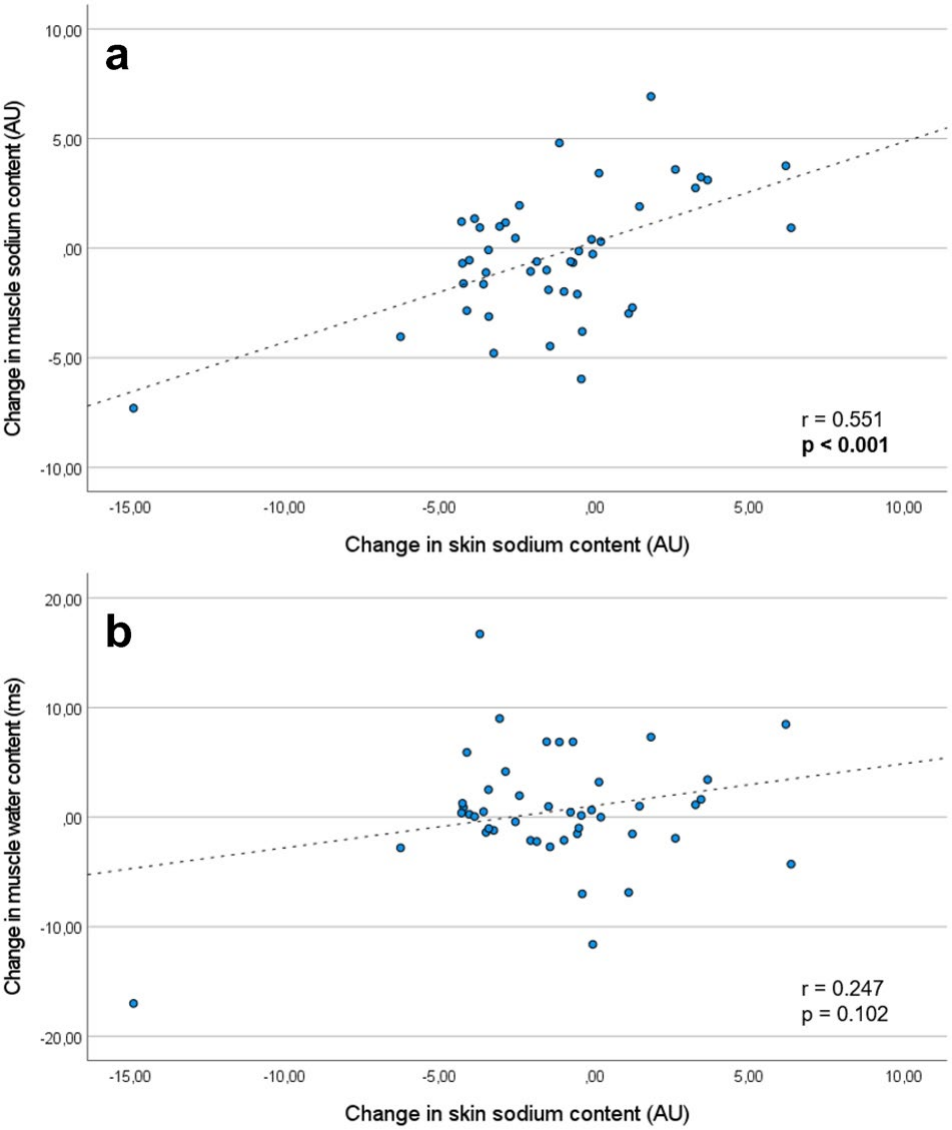
Relationship between change in skin sodium content and change in muscle sodium content **a** as well as change in muscle water content, **b** after 3 months treatment with empagliflozin. *After ignoring the potential outlier (left lower corner of the graph), the correlation between change in muscle sodium content and change in skin sodium content was still significant (*r* = 0.445, *p* = 0.002) and the correlation between change in muscle water and change in skin sodium content was still not significant (*r* = − 0.066, *p* = 0.673)

After 1 month treatment with empagliflozin, we observed a significant correlation between the change in skin sodium content and the change in muscle sodium content (*r* = 0.661, *p* < 0.001), muscle water content (*r* = 0.413, *p* = 0.004), OH (*r* = 0.665, *p* < 0.001) and ratio OH/extracellular water (*r* = 0.677, *p* < 0.001). After 3 months of treatment with empagliflozin, a significant correlation between the change in skin sodium content and the change in muscle sodium content (*r* = 0.551, *p* < 0.001) (Fig. 5a), OH (*r* = 0.474, *p* = 0.002) and ratio OH/extracellular water (*r* = 0.512, *p* = 0.001) persisted. Of note, the correlation between the change in skin sodium content and the change in muscle water content was no longer evident (Fig. 5b).

No relationship was found between the change in haematocrit, 24-h urine volume excretion, 24-h urine sodium or urine glucose excretion, body weight and BMI and either the change in skin sodium content nor the change in muscle sodium content after 1 and 3 months treatment with empagliflozin (data not given).

## Discussion

Randomised clinical trials conducted with empagliflozin or dapagliflozin in patients with CHF have demonstrated protective effects of SGLT2 inhibitors concerning development of heart failure, incident heart failure hospitalisations, all-cause mortality and renal failure [2–4]. To our knowledge, our work is the first trial to analyse the effect of the SGLT2 inhibitor empagliflozin on tissue sodium content, assessed non-invasively by ^23^Na-MRI, in euvolemic patients with CHF under stable conditions. The SGLT2 inhibitor empagliflozin was capable to reduce skin sodium content as well as bone marrow sodium content in our patients with stable CHF compared to baseline as well as to placebo after 1 and 3 months of treatment. No significant change in muscle water and muscle fat content was observed after 1 month and 3 months compared to baseline in the empagliflozin and in the placebo group as well as in direct comparison of both groups.

These results are in accordance with the results of a small exploratory analysis showing a significant reduction in skin sodium content with no reduction in muscle sodium and muscle water content in patients with T2D after 6 weeks treatment with dapagliflozin [22]. In patients hospitalised with acute heart failure a large decrease in skin sodium content of − 11.3 ± 2.5 AU (*p* < 0.001) and in muscle sodium content of − 6.5 ± 0.3 AU (*p* < 0.05) with no significant change in muscle water content (*p* = 0.17) was observed under intravenous furosemide therapy, but these results are not comparable to our current results [23]. We examined patients with no clinical signs of congestion and they were under stable oral diuretic therapy with no change of the diuretic dose or other concomitant medication during the trial. We analysed potential influencing parameters on change in skin sodium content such as treatment with mineralocorticoid receptor antagonist, gender, weight and systolic BP change and found no confounding effect. The precise underlying mechanism of the observed reduction in tissue sodium content under treatment with SGLT2 inhibitors is not yet clearly specified.

With the current MRI technique, we are not able to accurately differentiate between intracellular and extracellular interstitial sodium ion signals and thus to separate whether the observed decrease in skin sodium content is indicative of reduced micro-oedema or/and reduced water-independent bound sodium or both (and this is most likely the case in our opinion). The muscle inversion recovery ^23^Na density adapted sequence was a first attempt to separate the sodium signal, but still needs further development. The resolution of the current MRI technique, resulting in partial volume effects, allows only an absolutely accurate quantification of extravascular sodium ions from intravascular sodium ions.

Indirect signs of our trial, supporting the fact that the measured change in skin sodium content is related to the non-osmotic sodium, are the absence of a correlation between change in skin sodium content and change in muscle water content as well as in 24-h urine volume excretion, 24-h urine sodium excretion and 24-h urine glucose excretion. However, a close relationship between change in skin sodium content and change in muscle sodium content was observed after 1 and 3 months treatment with empagliflozin, indicating a parallel line in the changes. Possible explanations for the lack of significant difference in change in muscle sodium content are either smaller and more heterogenous changes in the muscle than in the skin or the short follow-up. Further, there is a relation between change in muscle sodium content and muscle water content that persisted after 3 months. These observations may reflect the glycoaminoglycan bound sodium in the tissue. Others have shown that in patients on haemodialysis, sodium content in muscle (*p* < 0.01) was reduced by fluid removal via haemodialysis, whereas sodium content in the skin (*p* = 0.1) only tended to be lower [24]. Sodium reduction may be due to an osmotic shift in the muscle as indicated by the correlation plot, however skin sodium reduction seems to be at least to some extent water-independent.

Increased tissue sodium content may lead, in addition to micro-oedema, to cardiac as well as vascular effects, which ultimately lead to CHF. In experimental and in vivo studies, increased tissue sodium content (high sodium concentration in the extracellular fluid) lead to increased hypertrophic signalling, e.g. induced directly hypertrophic responses in myocardial myoblasts and vascular smooth muscle cells of neonatal rats [25]. Left ventricular hypertrophy constitutes an independent predictor of cardiovascular morbidity, and in patients with reduced eGFR, skin sodium content of the lower leg closely modulated the extent of left ventricular mass increase, independent of confounding factors such as 24-h ambulatory BP or total body hydration [26]. Likewise, vascular hypertrophy leads to arterial stiffness and consequently to increased afterload that represents the hemodynamic pathway towards CHF [27]. In patients with T2D, parameters describing hypertrophic remodeling of retinal arterioles like wall thickness and cross sectional area are related to skin and muscle sodium content, independently of other confounding factors such as age, sex and 24-h ambulatory BP [28]. A reduction of tissue sodium content may reverse the above-described direct hypertrophic effects of sodium on the vascular smooth muscle and myocardial cell leading to a decrease in afterload and left ventricular hypertrophy. In addition to trophic effects of sodium, increased tissue sodium content leads to an enhanced response to endogenous vasoconstrictive hormones. Under experimental conditions, pre-capillary skin arterioles of rats on a high-salt diet showed increased hormonal vasoreactivity towards the circulating hormone angiotensin II [29]. This up-regulation of hormonal sensitivity leads to increased peripheral vascular resistance and consequently increased afterload imposed on the left ventricle.

It has been demonstrated that patients with CHF have myocardial interstitial oedema, which causes systolic and diastolic cardiac dysfunction and increased stiffness of the left ventricle [30]. Opposite to loop diuretics, which lead besides reduction of interstitial fluid, simultaneously to a reduction of intravascular volume, SGLT2 inhibitors reduce selectively interstitial fluid with only a minimal change in blood volume [31, 32]. Such a decrease in myocardial oedema may contribute to the improved prognosis observed in patients with CHF started on SGLT2 inhibitors. In the empagliflozin group, the observed decrease in skin sodium content was related to a decrease in overhydration measured by bioimpedance spectroscopy and a decrease in muscle water T2 value assessed by ^23^Na-MRI. The observed correlations between reduction in muscle sodium content and reduction in OH support the conclusion that SGLT2 inhibitors reduce micro-oedema. Recently, a reduction of myocardial extracellular volume, that is of myocardial micro-oedema, was observed in patients with T2D and coronary artery disease after 6 months treatment with empagliflozin, accompanied with a reduction of left ventricular mass [33]. The reverse remodeling may be driven by a reduction in extracellular, interstitial volume or intracellular volume (cardiomyocyte mass), or even both [33]. The reduction of myocardial micro-oedema is associated with an improvement of the compromised cardiac function and thereby with an improved prognosis [34].

We showed reduced office BP and improved metabolic control, findings well described in our previous studies [22, 35]. In a previously published analysis that focused on vascular effects in patients with CHF, we showed a significant decrease in central systolic BP and central pulse pressure under resting conditions as well as under 24-h ambulatory conditions after 1 and 3 months of treatment with empagliflozin [17]. These effects are mediated by the reduction of the preload through natriuresis and osmotic diuresis as well as of the afterload through reducing the arterial stiffness (indicated by the decrease in central BP and improving vascular function [17, 31].

The broad known mode of action of SGLT2 inhibitors is enhanced glucosuria and natriuresis. Initially at 1 month, a significant increase in 24 h urinary sodium excretion was observed, as already by others, which was although no longer evident at 3 months and may explain in part the rapid onset of reduced incidence of heart failure hospitalisation observed in the prospective endpoint trial [3, 36]. The initial natriuresis and polyuria in connection to reduction in extracellular water and in BP result in the activation of the renin-angiotensin system, leading to a transiently increase of renin activity in the first month after starting SGLT2 inhibitors. After 3 months a new steady state is reached due to this compensatory mechanisms leading to sodium reabsorption at other nephron sites, explaining the adjournment of 24-h urinary sodium excretion.

We think that the changes in bone marrow sodium content mirror the changes observed in the skin and constitute a finding of unclear significance. Furthermore, the measurement of bone marrow sodium content lacks validation against a gold standard method as shown for skin and muscle sodium content [8].

Several limitations should be pointed out. This was a single-center trial with an overall small sample size. However, this trial was not designed to be a clinical outcome trial, but a mechanistic trial. The chosen sample size was based on power calculation according to the data of our previous trial [22]. The high quality of this trial is underlined by the double-blind and randomised design. Secondly, we used bioimpedance measurements to assess the volume status of our patients, a technology often criticised. However recently, a close relationship between ^23^Na-MRI and bioimpedance measurements was observed in patients with chronic kidney disease stage 5. In these patients, tissue sodium content of muscle and skin correlated with bioimpedance measurements having an inverse linear relationship to extracellular resistance [37]. Third, our results cannot be extrapolated to patients with heart failure and preserved ejection fraction (HFpEF), constituting a relevant part of patients with heart failure, as the focus was based on patients with HFrEF or HFmrEF.

## Conclusion

We found a significant reduction in tissue sodium content after 1 and 3 months treatment with the SGLT2 inhibitor empagliflozin. Our data support the concept that increased skin sodium content in patients with CHF has clinically relevant pathogenic effects, leading to cardiac as well as vascular dysfunction and consequently to CHF. The reduction of extravascular sodium content may reflect a decrease in subclinical micro-oedema or/and in non-osmotic bound sodium, both reported to impair left ventricular function.

## Supplementary Information

Below is the link to the electronic supplementary material.

Supplementary file1 (DOCX 192 KB)

## Abbreviations

^23^Na-MRI: Sodium-MRI
AU: Arbitrary units
BCM: Body composition monitor
BP: Blood pressure
CHF: Chronic heart failure
eGFR: Estimated glomerular filtration rate
FMD: Flow mediated vasodilatation
HbA1c: Glycated haemoglobin
HFmrEF: Heart failure with mid-range ejection fraction
HFrEF: Heart failure with reduced ejection fraction
NTproBNP: N-terminal pro-brain natriuretic peptide
NYHA: New York Heart Association
OH: Overhydration
RF: Radiofrequency
SGLT2: Sodium-glucose co-transporter 2
T2D: Type 2 diabetes mellitus

## References

[1] Gallo LA, Wright EM, Vallon V (2015). Probing SGLT2 as a therapeutic target for diabetes: basic physiology and consequences. Diab Vasc Dis Res.

[2] McMurray JJV, Solomon SD, Inzucchi SE, Kober L, Kosiborod MN, Martinez FA, Ponikowski P, Sabatine MS, Anand IS, Belohlavek J, Bohm M, Chiang CE, Chopra VK, de Boer RA, Desai AS, Diez M, Drozdz J, Dukat A, Ge J, Howlett JG, Katova T, Kitakaze M, Ljungman CEA, Merkely B, Nicolau JC, O’Meara E, Petrie MC, Vinh PN, Schou M, Tereshchenko S, Verma S, Held C, DeMets DL, Docherty KF, Jhund PS, Bengtsson O, Sjostrand M, Langkilde AM, Committees D-HT, Investigators (2019). Dapagliflozin in patients with heart failure and reduced ejection fraction. N Engl J Med.

[3] Packer M, Anker SD, Butler J, Filippatos G, Pocock SJ, Carson P, Januzzi J, Verma S, Tsutsui H, Brueckmann M, Jamal W, Kimura K, Schnee J, Zeller C, Cotton D, Bocchi E, Bohm M, Choi DJ, Chopra V, Chuquiure E, Giannetti N, Janssens S, Zhang J, Gonzalez Juanatey JR, Kaul S, Brunner-La Rocca HP, Merkely B, Nicholls SJ, Perrone S, Pina I, Ponikowski P, Sattar N, Senni M, Seronde MF, Spinar J, Squire I, Taddei S, Wanner C, Zannad F, Investigators EM-RT (2020). Cardiovascular and renal outcomes with empagliflozin in heart failure. N Engl J Med.

[4] Packer M, Butler J, Zannad F, Filippatos G, Ferreira JP, Pocock SJ, Carson P, Anand I, Doehner W, Haass M, Komajda M, Miller A, Pehrson S, Teerlink JR, Schnaidt S, Zeller C, Schnee JM, Anker SD (2021). Effect of empagliflozin on worsening heart failure events in patients with heart failure and preserved ejection fraction: EMPEROR-preserved trial. Circulation.

[5] Herrington WG, Savarese G, Haynes R, Marx N, Mellbin L, Lund LH, Dendale P, Seferovic P, Rosano G, Staplin N, Baigent C, Cosentino F (2021). Cardiac, renal, and metabolic effects of sodium-glucose co-transporter-2 inhibitors: a position paper from the European Society of Cardiology ad-hoc task force on sodium-glucose co-transporter-2 inhibitors. Eur J Heart Fail.

[6] Marx N, McGuire DK (2016). Sodium-glucose cotransporter-2 inhibition for the reduction of cardiovascular events in high-risk patients with diabetes mellitus. Eur Heart J.

[7] Tager T, Frankenstein L, Atar D, Agewall S, Frey N, Grundtvig M, Clark AL, Cleland JGF, Frohlich H (2022). Influence of receptor selectivity on benefits from SGLT2 inhibitors in patients with heart failure: a systematic review and head-to-head comparative efficacy network meta-analysis. Clin Res Cardiol.

[8] Kopp C, Linz P, Wachsmuth L, Dahlmann A, Horbach T, Schofl C, Renz W, Santoro D, Niendorf T, Muller DN, Neininger M, Cavallaro A, Eckardt KU, Schmieder RE, Luft FC, Uder M, Titze J (2012). (23)Na magnetic resonance imaging of tissue sodium. Hypertension.

[9] Titze J, Shakibaei M, Schafflhuber M, Schulze-Tanzil G, Porst M, Schwind KH, Dietsch P, Hilgers KF (2004). Glycosaminoglycan polymerization may enable osmotically inactive Na+ storage in the skin. Am J Physiol Heart Circ Physiol.

[10] Hofmeister LH, Perisic S, Titze J (2015). Tissue sodium storage: evidence for kidney-like extrarenal countercurrent systems?. Pflugers Arch.

[11] Wiig H, Luft FC, Titze JM (2018). The interstitium conducts extrarenal storage of sodium and represents a third compartment essential for extracellular volume and blood pressure homeostasis. Acta Physiol (Oxf).

[12] Olde Engberink RH, Rorije NM, van den Born BH, Vogt L (2017). Quantification of nonosmotic sodium storage capacity following acute hypertonic saline infusion in healthy individuals. Kidney Int.

[13] Zaric O, Juras V, Szomolanyi P, Schreiner M, Raudner M, Giraudo C, Trattnig S (2021). Frontiers of sodium MRI revisited: from cartilage to brain imaging. J Magn Reson Imaging.

[14] Hu R, Kleimaier D, Malzacher M, Hoesl MAU, Paschke NK, Schad LR (2020). X-nuclei imaging: current state, technical challenges, and future directions. J Magn Reson Imaging.

[15] Kolwelter J, Uder M, Schmieder RE (2020). Tissue sodium content in hypertension and related organ damage. J Hypertens.

[16] Christa M, Weng AM, Geier B, Wormann C, Scheffler A, Lehmann L, Oberberger J, Kraus BJ, Hahner S, Stork S, Klink T, Bauer WR, Hammer F, Kostler H (2019). Increased myocardial sodium signal intensity in Conn’s syndrome detected by 23Na magnetic resonance imaging. Eur Heart J Cardiovasc Imaging.

[17] Kolwelter J, Bosch A, Jung S, Stabel L, Kannenkeril D, Ott C, Bramlage P, Schiffer M, Achenbach S, Schmieder RE (2021). Effects of the sodium-glucose cotransporter 2 inhibitor empagliflozin on vascular function in patients with chronic heart failure. ESC Heart Fail.

[18] Williams B, Mancia G, Spiering W, Rosei EA, Azizi M, Burnier M, Clement DL, Coca A, de Simone G, Dominiczak A, Kahan T, Mahfoud F, Redon J, Ruilope L, Zanchetti A, Kerins M, Kjeldsen SE, Kreutz R, Laurent S, Lip GYH, McManus R, Narkiewicz K, Ruschitzka F, Schmieder RE, Shlyakhto E, Tsioufis C, Aboyans V, Desormais I, Grp ESD (2019). 2018 ESC/ESH Guidelines for the management of arterial hypertension (vol 39, pg 3021, 2018). Eur Heart J.

[19] Moissl UM, Wabel P, Chamney PW, Bosaeus I, Levin NW, Bosy-Westphal A, Korth O, Muller MJ, Ellegard L, Malmros V, Kaitwatcharachai C, Kuhlmann MK, Zhu F, Fuller NJ (2006). Body fluid volume determination via body composition spectroscopy in health and disease. Physiol Meas.

[20] Chamney PW, Wabel P, Moissl UM, Muller MJ, Bosy-Westphal A, Korth O, Fuller NJ (2007). A whole-body model to distinguish excess fluid from the hydration of major body tissues. Am J Clin Nutr.

[21] Bland JAD (1986). Statistical methods for assessing agreement between two methods of clinical measurement. Lancet.

[22] Karg MV, Bosch A, Kannenkeril D, Striepe K, Ott C, Schneider MP, Boemke-Zelch F, Linz P, Nagel AM, Titze J, Uder M, Schmieder RE (2018). SGLT-2-inhibition with dapagliflozin reduces tissue sodium content: a randomised controlled trial. Cardiovasc Diabetol.

[23] Hammon M, Grossmann S, Linz P, Kopp C, Dahlmann A, Garlichs C, Janka R, Cavallaro A, Luft FC, Uder M, Titze J (2015). 23Na magnetic resonance imaging of the lower leg of acute heart failure patients during diuretic treatment. PLoS One.

[24] Dahlmann A, Dorfelt K, Eicher F, Linz P, Kopp C, Mossinger I, Horn S, Buschges-Seraphin B, Wabel P, Hammon M, Cavallaro A, Eckardt KU, Kotanko P, Levin NW, Johannes B, Uder M, Luft FC, Muller DN, Titze JM (2015). Magnetic resonance-determined sodium removal from tissue stores in hemodialysis patients. Kidney Int.

[25] Gu J, Anand V (1998). Sodium induces hypertrophy of cultured myocardial myoblasts and vascular smooth muscle cells. Hypertension.

[26] Schneider MP, Raff U, Kopp C, Scheppach JB, Toncar S, Wanner C, Schlieper G, Saritas T, Floege J, Schmid M, Birukov A, Dahlmann A, Linz P, Janka R, Uder M, Schmieder RE, Titze JM, Eckardt KU (2017). Skin sodium concentration correlates with left ventricular hypertrophy in CKD. J Am Soc Nephrol.

[27] Pandey A, Khan H, Newman AB, Lakatta EG, Forman DE, Butler J, Berry JD (2017). Arterial stiffness and risk of overall heart failure, heart failure with preserved ejection fraction, and heart failure with reduced ejection fraction: the health ABC Study (health, aging, and body composition). Hypertension.

[28] Schmieder R, Jung S, Kannenkeril D, Harazny JM, Striepe K, Ott C, Linz P, Nagel AM, Uder M (2019). P4993Tissue sodium concentration emerged as a determinant of hypertrophic vascular remodeling in type 2 diabetes. Eur Heart J.

[29] Helle F, Karlsen TV, Tenstad O, Titze J, Wiig H (2013). High-salt diet increases hormonal sensitivity in skin pre-capillary resistance vessels. Acta Physiol (Oxf).

[30] Davis KL, Mehlhorn U, Laine GA, Allen SJ (1995). Myocardial edema, left ventricular function, and pulmonary hypertension. J Appl Physiol (1985).

[31] Verma S, McMurray JJV (2018). SGLT2 inhibitors and mechanisms of cardiovascular benefit: a state-of-the-art review. Diabetologia.

[32] Fujiki S, Tanaka A, Imai T, Shimabukuro M, Uehara H, Nakamura I, Matsunaga K, Suzuki M, Kashimura T, Minamino T, Inomata T, Node K, Investigators CT (2022). Body fluid regulation via chronic inhibition of sodium-glucose cotransporter-2 in patients with heart failure: a post hoc analysis of the CANDLE trial. Clin Res Cardiol.

[33] Mason T, Coelho-Filho OR, Verma S, Chowdhury B, Zuo F, Quan A, Thorpe KE, Bonneau C, Teoh H, Gilbert RE, Leiter LA, Juni P, Zinman B, Jerosch-Herold M, Mazer CD, Yan AT, Connelly KA (2021). Empagliflozin reduces myocardial extracellular volume in patients with type 2 diabetes and coronary artery disease. JACC Cardiovasc Imaging.

[34] Verbrugge FH, Bertrand PB, Willems E, Gielen E, Mullens W, Giri S, Tang WHW, Raman SV, Verhaert D (2017). Global myocardial oedema in advanced decompensated heart failure. Eur Heart J Cardiovasc Imaging.

[35] Striepe K, Jumar A, Ott C, Karg MV, Schneider MP, Kannenkeril D, Schmieder RE (2017). Effects of the selective sodium-glucose cotransporter 2 inhibitor empagliflozin on vascular function and central hemodynamics in patients with type 2 diabetes mellitus. Circulation.

[36] Zinman B, Lachin JM, Inzucchi SE (2016). Empagliflozin, cardiovascular outcomes, and mortality in type 2 diabetes. N Engl J Med.

[37] Mitsides N, McHugh D, Swiecicka A, Mitra R, Brenchley P, Parker GJM, Mitra S (2020). Extracellular resistance is sensitive to tissue sodium status; implications for bioimpedance-derived fluid volume parameters in chronic kidney disease. J Nephrol.

