# Supplementary material for: The SGLT2 inhibitor empagliflozin reduces tissue sodium content in patients with chronic heart failure: results from a placebo-controlled randomised trial
Source: Clin Res Cardiol. 2022 Oct 26;112(1):134–44. doi: 10.1007/s00392-022-02119-7 (PMC9849317; doi:10.1007/s00392-022-02119-7)
Supplement: Supplementary file 1 — Supplementary file1 (DOCX 192 KB) [file 392_2022_2119_MOESM1_ESM.docx]

The SGLT2 inhibitor empagliflozin reduces tissue sodium content in patients with chronic heart failure: results from a placebo-controlled randomised trial

*– Clinical Research in Cardiology –*

**SUPPLEMENTARY INFORMATION**

**Authors:** Julie Kolwelter^1,2^, Dennis Kannenkeril^1^, Peter Linz^1,3^, Susanne Jung^2^, Armin M. Nagel^3,4^, Agnes Bosch^1^, Christian Ott^1,5^, Peter Bramlage^6^, Lisa Nöh^1^, Mario Schiffer^1^, Michael Uder^3^, Stephan Achenbach^2^, Roland E. Schmieder^1^

**Affiliations:**^1^Department of Nephrology and Hypertension, University Hospital Erlangen, Friedrich-Alexander University Erlangen-Nuremberg (FAU), Germany
^2^Department of Cardiology, University Hospital Erlangen, Friedrich-Alexander University Erlangen-Nuremberg (FAU), Germany
^3^Institute of Radiology, University Hospital Erlangen, Friedrich-Alexander University Erlangen-Nuremberg (FAU), Germany
^4^Division of Medical Physics in Radiology, German Cancer Research Center (DKFZ), Heidelberg, Germany
^5^Department of Nephrology and Hypertension, Paracelsus Medical University, Nuremberg, Germany
^6^Institute for Pharmacology and Preventive Medicine, Cloppenburg, Germany

**Address for Correspondence**

Prof. Dr. Roland E. Schmieder

Head of the Clinical Research Center

Department of Nephrology and Hypertension, University Hospital Erlangen

Friedrich-Alexander University Erlangen-Nuremberg (FAU)

Ulmenweg 18, 91054 Erlangen, Germany

Phone: +49 9131 85 39002

Fax: +49 9131 85 39209

E-Mail: [Roland.Schmieder@uk-erlangen.de](mailto:Roland.Schmieder@uk-erlangen.de)

**Supplemental methods**

**^23^Na-MRI**

^23^Na-MRI requires larger voxel volumes and longer acquisition times compared to conventional ^1^H-MRI. This is due to the low in vivo concentration of sodium ions and 10-fold lower nuclear magnetic resonance sensitivity of sodium. Once excited, the sodium nucleus possesses very short relaxation times, so that sequences with ultrashort echo time are necessary to enable quantitative measurements.[1] ^23^Na-MRI was performed with a Cartesian 2D gradient echo (GRE) sequence: total acquisition time 13:41 min, echo time 2.1 ms, repetition time 100 ms, flip angle 90°, 128 averages, resolution 3 mm x 3 mm x 30 mm. To estimate the amount of bound sodium ions, a relaxation-weighted, inversion recovery, ^23^Na density adapted 3D radial sequence was applied to suppress the sodium signal originating from free (liquid, water-bound) sodium ions such as in the interstitial space: total acquisition time 9:50 min, echo time 0.3 ms, repetition time 124 ms, inversion time 34 ms, flip angle 90°, nominal resolution 4 mm x 4 mm x 20 mm.[2] The calibration tubes with agarose gel (two from four 60ml HDPE Nalgene® flasks filled with 20 and 40 mmol/l NaCl with 5% agarose gel) placed in the phantom holder underneath the calf are used to attempt to quantify non-osmotic bound sodium ions. In parallel, conventional ^1^H-images were acquired. To estimate muscle fat content, a T1 weighted 6-point VIBE Q-DIXON sequence was used: total acquisition time 4:00 min, echo times 1.52, 3.07, 4.88, 6.71, 8.54 and 10.37 ms, repetition time 50 ms, flip angle 6°, resolution 1.5 mm.

To estimate muscle water content, T2 maps were acquired with the body coil of the scanner using a spin-echo sequence: total acquisition 6:29 min, echo time 9.5 ms, 340 ms using 32 echos, repetition time 3 ms, resolution 1.5 mm x 1.5 mm x 10 mm. The first echo of this sequence served as anatomically orientation for defining regions of interest (ROI) for triceps muscle and tibial bone marrow. ROIs in the skin were defined where the calf was in direct contact with the cylindrical surface of the phantom holder to reduce inter-individual differences in geometries and a layer thickness of one pixel was evaluated in the ^23^Na-GRE image. The intensities of the acquired greyscale regions and concentrations in the calibration tubes were then analysed in a linear regression curve and used to extrapolate sodium content.

*Supplemental References*

1. Konstandin S, Nagel AM. Measurement techniques for magnetic resonance imaging of fast relaxing nuclei. MAGMA. 2014;27(1):5-19.

2. Nagel AM, Amarteifio E, Lehmann-Horn F, Jurkat-Rott K, Semmler W, Schad LR, Weber MA. 3 Tesla sodium inversion recovery magnetic resonance imaging allows for improved visualization of intracellular sodium content changes in muscular channelopathies. Invest Radiol. 2011;46(12):759-66.

**Supplemental Results**

**Supplemental Fig 1** Patient disposition


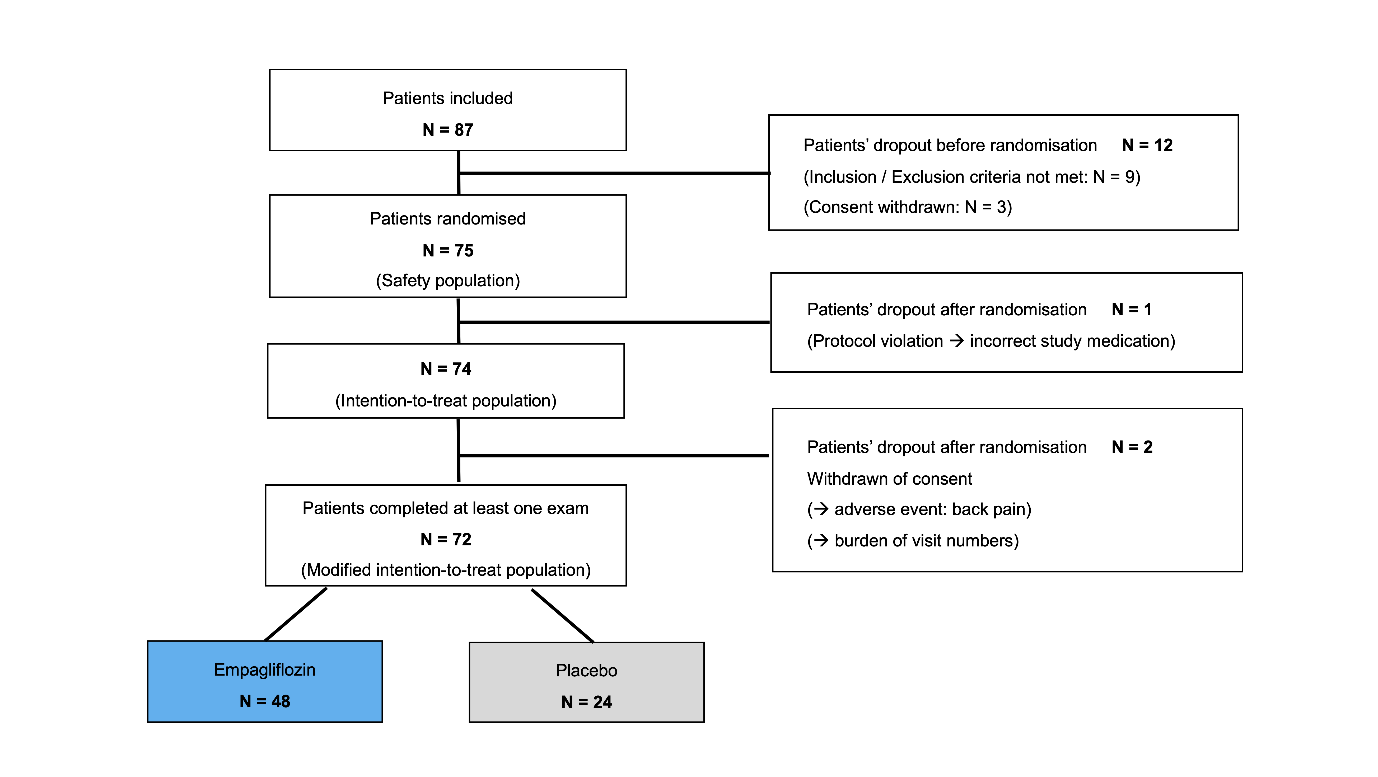


**Supplemental Table 1** Changes in ^23^NaMRI parameters after 1 month of treatment with empagliflozin or placebo

| **Parameters**  **after 1 month** | **Empagliflozin,**  **change from baseline** | **P-value**  **empagliflozin vs. baseline** | **Placebo**  **change from baseline** | **P-value**  **placebo vs. baseline** | **Between- group difference** | **P-value**  **empagliflozin vs. placebo** |
| --- | --- | --- | --- | --- | --- | --- |
| Skin sodium content (AU) | -1.2 ± 3.9 | 0.039 | -0.3 ± 3.5 | 0.658 | -0.8 ± 1.0 | 0.411 |
| Muscle sodium content (AU) | -0.7 ± 3.2 | 0.138 | -1.1 ± 2.5 | 0.061 | 0.4 ± 0.8 | 0.664 |
| Muscle sodium content, inversion recovery sequence (AU) | 0.0 ± 1.8 | 0.904 | -0.8 ± 3.4 | 0.314 | -0.8 ± 0.7 | 0.322 |
| Muscle water content (ms) | 0.5 ± 5.1 | 0.550 | -0.8 ± 4.1 | 0.369 | 1.3 ± 1.2 | 0.317 |
| Muscle fat content (AU) | 0.2 ± 1.5 | 0.346 | 0.4 ± 2.7 | 0.508 | -0.2 ± 0.5 | 0.716 |
| Tibial bone marrow sodium content (AU) | -0.5 ± 1.2 | 0.010 | 0.1 ± 1.4 | 0.855 | -0.6 ± 0.3 | 0.110 |

Data are mean ± standard deviation. AU, arbitrary units.

**Supplemental Table 2** Changes in clinical parameters after 1 month of treatment with empagliflozin or placebo

| **Parameters**  **after 1 month** | **Empagliflozin,**  **change from baseline** | **P-value**  **empagliflozin vs. baseline** | **Placebo, change from baseline** | **P-value**  **placebo vs. baseline** | **Between- group difference** | **P-value**  **empagliflozin vs. placebo** |
| --- | --- | --- | --- | --- | --- | --- |
| Weight (kg) | -0.4 ± 1.4 | 0.039 | 0.3 ± 1.5 | 0.381 | -0.7 ± 0.3 | 0.53 |
| BMI (kg/m^2^) | -0.1 ± 0.4 | 0.036 | 0.1 ± 0.5 | 0.369 | -0.2 ± 0.1 | 0.048 |
| Office SBP (mmHg) | -8.9 ± 13.3 | <0.001 | -0.1 ± 11.1 | 0.967 | -8.8 ± 3.1 | 0.006 |
| Office DBP (mmHg) | -3.4 ± 7.4 | 0.002 | 0.6 ± 8.2 | 0.712 | -4.1 ± 1.9 | 0.036 |
| Office heart rate (bpm) | -2.0 ± 12.6 | 0.272 | -1.2 ± 10.7 | 0.593 | -0.4 ± 3.4 | 0.908 |
| OH (l) | -0.26 ± 0.8 | 0.038 | -0.05 ± 1.0 | 0.822 | -0.21 ± 0.2 | 0.357 |
| OH/ECW (%) | -1.33 ± 4.5 | 0.046 | -0.26 ± 5.9 | 0.835 | -1.07 ± 1.3 | 0.401 |

Data are mean ± standard deviation. BMI, body mass index; SBP, systolic blood pressure; DBP, diastolic blood pressure; bpm, beats per minute; OH, overhydration; OH/ECW, ratio of overhydration and extracellular water.

**Supplemental Table 3** Changes in laboratory parameters after 1 month of treatment with empagliflozin or placebo

| **Parameters**  **after 1 month** | **Empagliflozin**  **change from baseline** | **P-value**  **empagliflozin vs. baseline** | **Placebo, change from baseline** | **P-value**  **placebo vs. baseline** | **Between- group difference** | **P-value**  **empagliflozin vs. placebo** |
| --- | --- | --- | --- | --- | --- | --- |
| NTproBNP (pg/ml) | -4 (-176, 43) | 0.159 | 33 (-139, 315) | 0.376 |  | 0.162 |
| ln NTproBNP | -0.1 ± 0.4 | 0.289 | -0.0 ± 0.5 | 0.788 | -0.0 ± 0.1 | 0.723 |
| HbA1c (%) | -0.1 ± 0.2 | 0.096 | -0.0 ± 0.2 | 0.911 | -0.0 ± 0.1 | 0.341 |
| FP-glucose (mg/dl) | -3.9 ± 9.2 | 0.005 | 4.3 ± 11.8 | 0.090 | -8.2 ± 2.5 | 0.002 |
| Haematocrit (%) | 0.7 ± 2.5 | 0.055 | -0.0 ± 2.2 | 0.978 | 0.7 ± 0.6 | 0.233 |
| Serum creatinine (mg/dl) | 0.07 ± 0.1 | <0.001 | 0.01 ± 0.1 | 0.425 | 0.06 ± 0.0 | 0.037 |
| eGFR (ml/min/1.73m2) | -4.5 ± 8.0 | <0.001 | -1.2 ± 4.6 | 0.198 | -3.3 ± 1.7 | 0.066 |
| Serum uric acid (mg/dl) | -1.4 ± 0.9 | <0.001 | -0.1 ± 0.7 | 0.386 | -1.2 ± 0.2 | <0.001 |
| Serum sodium (mmol/l) | -0.1 ± 1.8 | 0.629 | -0.0 ± 1.3 | 0.882 | -0.1 ± 0.4 | 0.835 |
| 24-h urinary volume excretion (ml) | 394 ± 643 | <0.001 | 74 ± 596 | 0.548 | 319 ± 157 | 0.046 |
| 24-h urinary sodium excretion (mmol/24h) | 25.2 ± 81.9 | 0.040 | 2.8 ± 73.5 | 0.854 | 22.4 ± 19.9 | 0.263 |
| 24-h urinary glucose excretion (g/24h) | 34.5 ± 20.3 | <0.001 | 1.8 ± 7.9 | 0.310 | 32.7 ± 4.5 | <0.001 |
| 24-h urinary uric acid excretion (g/24h) | 0.09 ± 0.2 | <0.001 | 0.02 ± 0.2 | 0.594 | 0.07 ± 0.0 | 0.120 |

Data are mean ± standard deviation or median (interquartile range). NTproBNP, N-terminal prohormone of brain natriuretic peptide; ln, natural logarithm; HbA1c, glycated haemoglobin; FP-glucose, fasting-plasma glucose; eGFR, estimated glomerular filtration rate

**Supplemental Table 4** Changes in laboratory parameters after 3 months of treatment with empagliflozin or placebo

| **Parameters**  **after 3 months** | **Empagliflozin**  **change from baseline** | **P-value**  **empagliflozin vs. baseline** | **Placebo,**  **change from baseline** | **P-value**  **placebo vs. baseline** | **Between- group difference** | **P-value**  **empagliflozin vs. placebo** |
| --- | --- | --- | --- | --- | --- | --- |
| NTproBNP (pg/ml) | -17 (-2019, 65) | 0.125 | -41 (-207, 63) | 0.317 |  | 0.976 |
| ln NTproBNP | -0.1 ± 0.5 | 0.149 | -0.2 ± 0.6 | 0.100 | 0.1 ± 0.1 | 0.565 |
| HbA1c (%) | - 0.1 ± 0.3 | 0.023 | -0.0 ± 0.3 | 0.588 | -0.1 ± 0.1 | 0.378 |
| FP-glucose (mg/dl) | -6.6 ± 9.2 | <0.001 | 0.0 ± 8.7 | 0.981 | -6.6 ± 2.3 | 0.005 |
| Haematocrit (%) | 2.2 ± 2.3 | <0.001 | 0.5 ± 1.8 | 0.225 | 1.8 ± 0.5 | 0.002 |
| Serum creatinine (mg/dl) | 0.06 ± 0.1 | 0.002 | 0.04 ± 0.1 | 0.139 | 0.02 ± 0.0 | 0.465 |
| eGFR (ml/min/1.73m2) | -4.4 ± 9.8 | 0.003 | -2.1 ± 6.6 | 0.137 | -2.3 ± 2.2 | 0.240 |
| Serum uric acid (mg/dl) | -1.2 ± 0.9 | <0.001 | 0.1 ± 0.7 | 0.519 | -1.3 ± 0.2 | <0.001 |
| Serum sodium (mmol/l) | 0.3 ± 2.2 | 0.387 | 0.3 ± 1.4 | 0.246 | -0.1 ± 0.5 | 0.897 |
| 24-h urinary volume excretion (ml) | 222 ± 634 | 0.019 | 77 ± 815 | 0.649 | 145 ± 175 | 0.409 |
| 24-h urinary sodium excretion (mmol/24h) | 16.1 ± 82.5 | 0.187 | -9.1 ± 90.5 | 0.626 | 25.2 ± 21.4 | 0.242 |
| 24-h urinary glucose excretion (g/24h) | 35.8 ± 22.7 | <0.001 | -0.4 ± 1.4 | 0.171 | 36.2 ± 4.8 | <0.001 |
| 24-h urinary uric acid excretion (g/24h) | 0.03 ± 0.2 | 0.257 | 0.02 ± 0.2 | 0.652 | 0.01 ±0.0 | 0.805 |

Data are mean ± standard deviation or median (interquartile range). NTproBNP, N-terminal prohormone of brain natriuretic peptide; ln, natural logarithm; HbA1c, glycated haemoglobin; FP-glucose, fasting-plasma glucose; eGFR, estimated glomerular filtration rate
